# Arthroplasty implants and materials: Cost awareness and value perception

**DOI:** 10.1371/journal.pone.0255061

**Published:** 2021-07-26

**Authors:** Mursal Gardezi, Taylor D. Ottesen, Vineet Tyagi, Josiah J. Z. Sherman, Jonathan N. Grauer, Lee E. Rubin

**Affiliations:** 1 Yale School of Medicine, New Haven, CT, United States of America; 2 Department of Orthopaedics and Rehabilitation, New Haven, CT, United States of America; Azienda Ospedaliero Universitaria Pisana, ITALY

## Abstract

Arthroplasty procedures are commonly performed and contribute to healthcare expenditures seen in the United States. Surgical team members may make selections among implants and materials without always knowing their relative cost. The current study reports on a survey aimed to investigate the perceptions of an academic group about the relative cost and value of commonly used operating room implants and materials related to joint arthroplasty cases using 10 matched pairs of items. Of the 124 persons eligible to take the survey, 102 responded (response rate of 82.3%) including attendings, fellows, residents, physician assistants (PAs), advanced practice registered nurses (APRNs) and registered nurses (RNs). On average for the ten pairs of items, the more expensive items were correctly selected by 90.2+/-13.9% (mean+/- standard deviation) of respondents with a range from 54.9% to 100%. Of note, the cost differences were significantly overestimated for 8/10 item pairs. The majority of respondents perceived the more expensive item as the item with the higher clinical value for 9/10 item pairs. Most arthroplasty attendings (91.3%) indicated willingness to use the less expensive item of two similar items. Nonetheless, 17.9% of fellows, residents, PAs, APRNs and RNs indicated that they would not feel comfortable suggesting using the less expensive item. Although attending arthroplasty surgeons stated a desire to consider costs, a knowledge deficit with regards to identifying the extent of cost differences was identified, and a significant portion of the surgical support team reported being hesitant to suggest less expensive options.

## Introduction

Healthcare spending in the United States is the highest in the world [1] and is projected to increase to 6.2 trillion dollars by 2028 [2]. As one of the most commonly performed orthopaedic surgical procedures, total joint arthroplasty (TJA) constitutes one of the largest annual healthcare expenditures for the federal government [3, 4] with nearly 1.5 million cases occurring annually [5].

Costs related to TJA are substantial and can vary dramatically. For instance, the average implant cost used in total hip arthroplasty (THA) have been reported to range from $2,392 to $12,651 per case and may comprise up to 87% of the total surgical costs [6]. Similarly, implants used in total knee arthroplasty (TKA) have been reported to range from $1,797 to $12,093 [6].

Unlike other purchasing scenarios that are driven by market pressure, healthcare workers who select products do not directly pay for them and actually may not know the relative costs of what they are choosing between. Thus, even if being cost conscious, surgical teams may have difficulty effectively helping control costs [7].

There are a number of reasons healthcare workers do not know the actual costs of what they are using. To start, pricing agreements between suppliers and hospitals are often considered confidential and thus not always transparent to all clinical end users [8]. Also, negotiating power for items may differ amongst different hospitals, thus causing price variations between hospitals [8, 9]. Further, item prices may also change over time as contracts are renewed, novel products become commodities, etc. Finally, since physician reimbursements for patient care are typically not directly affected by item costs, physicians may lack strong incentives to learn about item costs [10].

Previous studies demonstrate that surgeons have not been able to predict cost well, both overestimating and underestimating costs [11, 12]. In contrast to predicting the cost of an item, the ability to differentiate a more expensive item of two choices may be a more clinically relevant measure of cost knowledge. One study asking surgeons to identify the more expensive item of two items found an average correct score of 66%, slightly higher than what is expected by chance [13]. In addition, not only the perceived cost difference, but also the perceived clinical value, of items may both be important in the choice of similar alternatives.

There are currently only a handful of studies that assess cost knowledge and decision making in surgery, many of which are limited to surgeons and residents and exclude other members of the surgical team [11–13]. In an effort to further elucidate the effect of cost awareness among members of the surgical team, the current study aimed to investigate the perceptions of those at an academic institution about the cost differences and perceived value of commonly used materials and implants that are regularly used for TJA in the operating rooms (ORs) where they work.

## Materials and methods

### Participants / survey

The current study was conducted between March and May 2020 at a single academic institution. All attending surgeons, fellows, residents, physician assistants (PAs), advanced practice registered nurses (APRNs), and registered nurses [9] in an academic department participating in the total joint replacement program of a hospital system were invited to participate.

The survey utilized in the current study was developed to assess perceptions of relative costs and value for arthroplasty related implants and materials. Items commonly used in arthroplasty cases were selected and matched with alternative items that achieved similar objectives. Items were chosen without knowledge of their relative or absolute costs by the investigators of this study. Respondents were presented with images and names of sets of two matched items and asked to estimate the relative cost and clinical value difference for ten sets of matched items.

The ten sets of items were randomized with regard to whether the more expensive item was presented first or second. Here, the higher cost item is presented first for each matched pair:

1. Cement gun versus cement bowl.
2. Antibiotic cement versus plain cement.
3. Silver impregnated dressing versus island dressing.
4. Cerclage cable versus cerclage wire.
5. Bipolar head for hemiarthroplasty versus unipolar head for hemiarthroplasty.
6. Oxinium femur versus cobalt chrome femur for Total Knee Arthroplasty.
7. Polyethylene total knee liner with vitamin E versus polyethylene total knee liner without vitamin E.
8. Delta ceramic versus cobalt chrome femoral head.
9. Mobile bearing unicompartmental knee arthroplasty versus fixed bearing unicompartmental knee arthroplasty.
10. Smoke evacuator electrocautery handle versus standard electrocautery handle.

Invitations were sent via email with a link to complete the electronic survey which was administered through Qualtrics (Provo, Utah), an electronic survey platform. Digital informed consent was obtained from participants before survey administration. Institutional review board (IRB) exemption status was granted for this study under Yale University IRB # 2000022419.

### Cost information / data analysis

Cost information was provided by the institution’s purchasing office. For the purposes of this survey, cost was defined as the amount, in dollars, the institution pays to vendors to purchase each item.

Data was imported into STATA. Fisher’s exact test was used to compare the percentage of correct responses in comparing matched items based on clinical role. T-tests were used to compare perceived cost differences with true cost differences between matched item pairs. Significance was defined as p < 0.05. Differences were assessed, as opposed to actual value, to avoid the concerns of disclosing confidential pricing and purchasing arrangements.

## Results

### Survey respondents and relative cost/value perceptions

**Fig 1:**
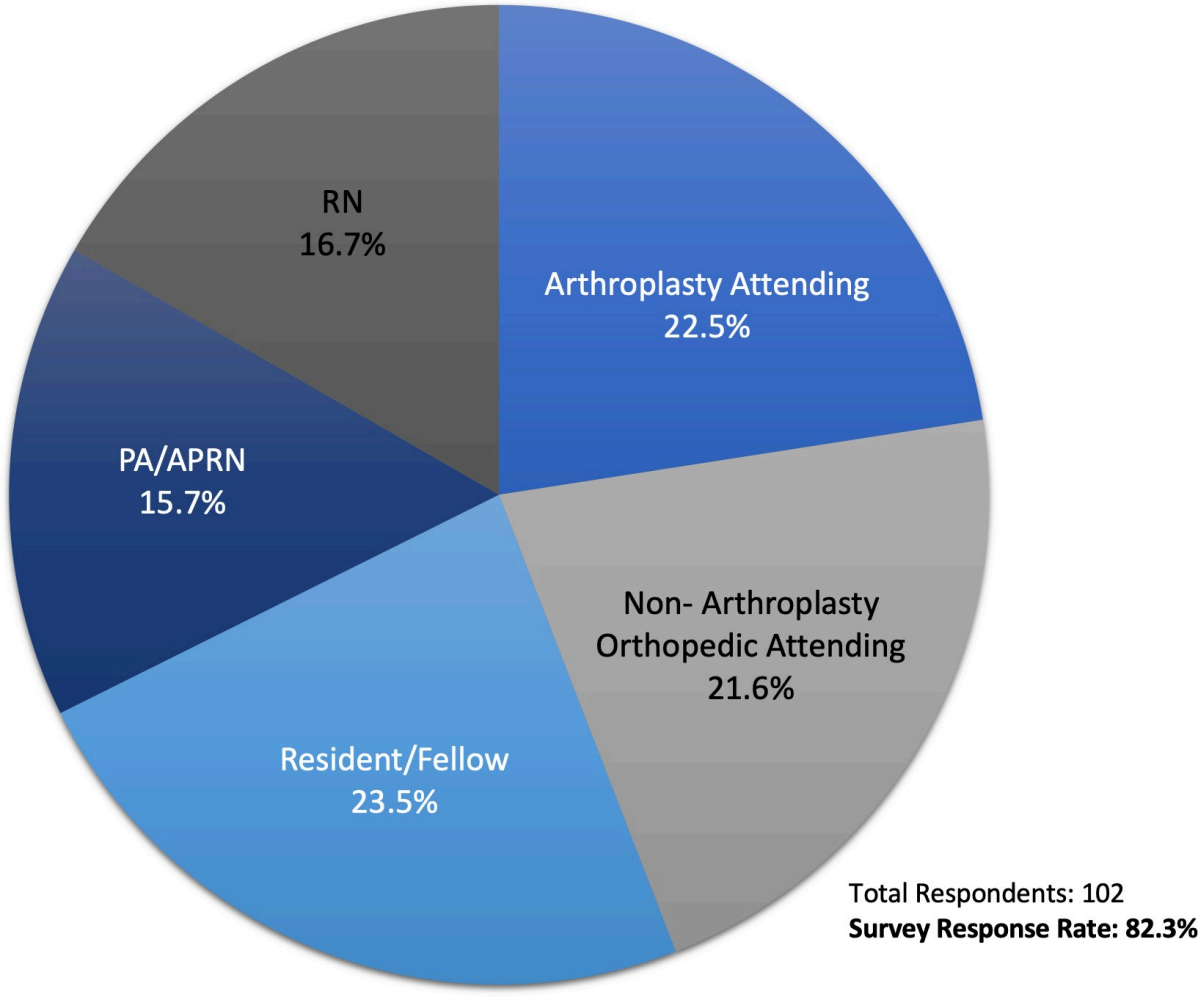
Survey respondents by training. Survey respondents by training level and role.

One hundred twenty-four people were invited to take part in the survey, of which 102 responded (response rate of 82.3%). Respondents included: arthroplasty attendings (22.5%), non-arthroplasty attendings (21.6%), residents/fellows (23.5%), PA/APRNs (15.7%), and RNs (16.7%) (Fig 1).

**Fig 2:**
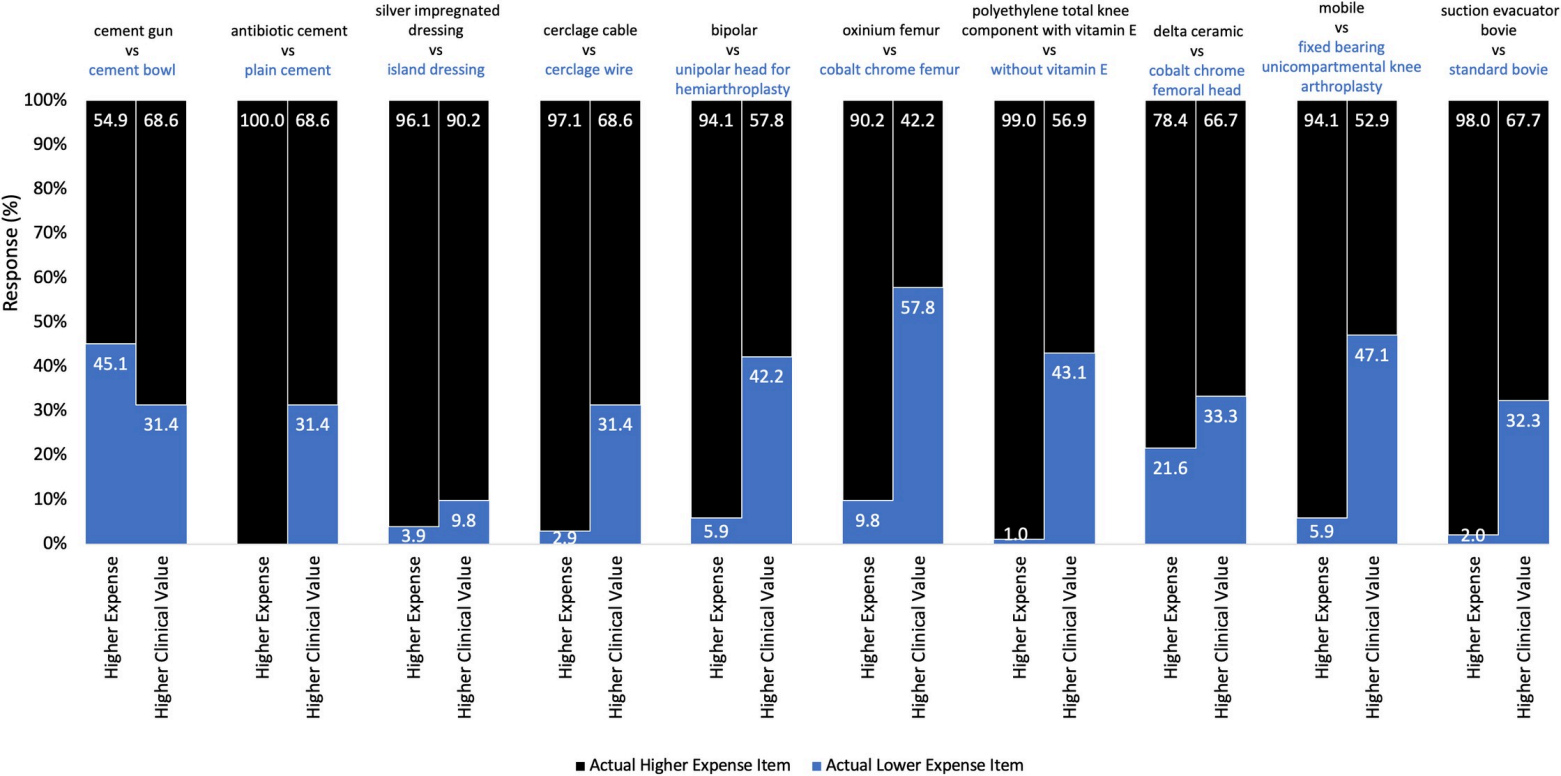
Perceived higher expense and clinical value between paired items. Perceived higher expense and clinical value between ten paired items for 102 respondents. Higher expense item of matched pairs underlined.

In terms of estimating the relative cost for the ten matched pairs of items, the respondents average +/- standard deviation correct by pair was 90.2 +/-13.9% (Fig 2). The percent accuracy for identifying the more expensive item for any given item pair had a large range: 54.9% to 100% correct.

Respondents were also asked to indicate the item in each pair they believed has the higher clinical value. The majority of respondents perceived the more expensive item as the item with the higher clinical value for nine out of the ten item pairs, but again with a large range (42.2% to 90.2%, Fig 2).

**Fig 3:**
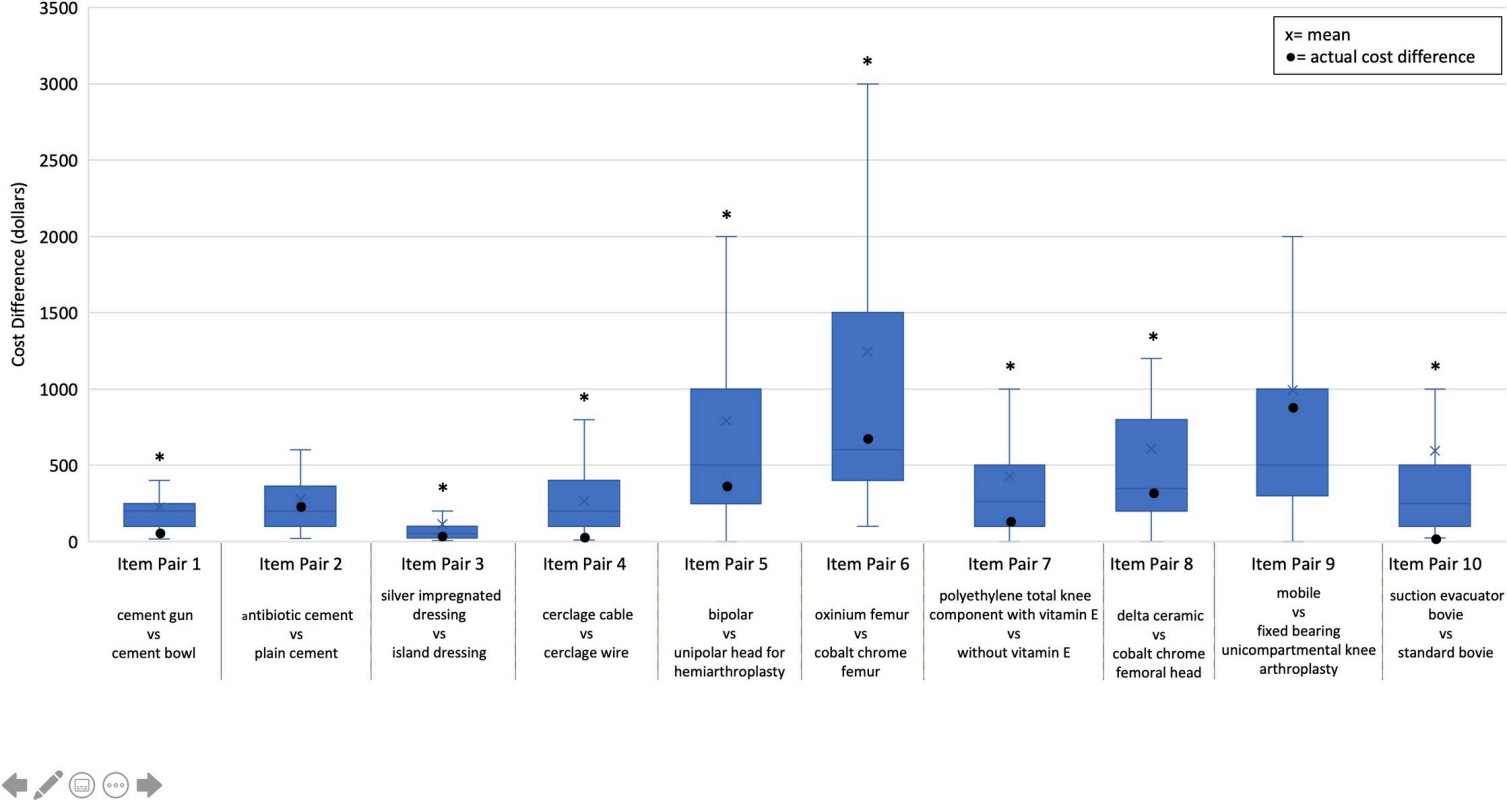
Perceived cost difference between paired items. Mean perceived cost differences between ten paired items for 102 respondents. x indicates the mean perceived cost difference, ● is the actual cost difference. T-tests used and significance (p< 0.05) is indicated by *.

Despite a relatively high rate of success in identifying an item as more expensive in the current study, clinicians poorly estimated the cost difference between the ten sets of matched items. The perceived cost differences were statistically higher than the actual cost difference for eight out of ten item pairs. The remaining two item pairs had mean perceived cost differences higher than the actual cost differences but did not reach statistical significance. Average cost difference estimates over the actual cost difference were as low as $43 and as high as $555 (Fig 3).

### Impact of cost difference perceptions

**Fig 4:**
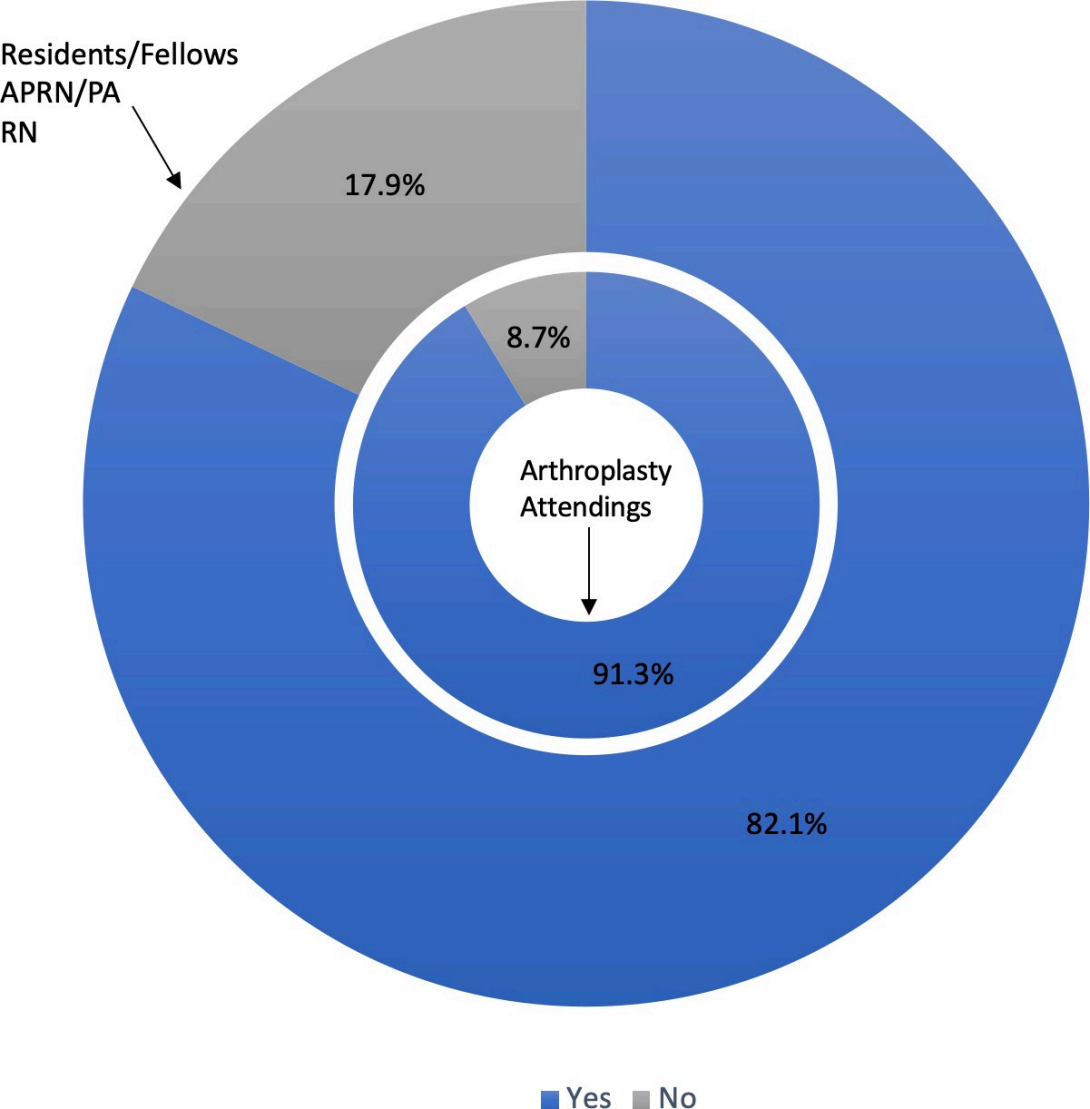
Clinicians willingness to use/suggest using a lower expense item. Clinicians indicated their willingness to use or suggest using a lower expense item if they were aware of cost differences of clinically similar items. The inner doughnut chart present results from arthroplasty attendings, while the outside doughnut chart presents results from all other respondents.

Currently, pricing information is not readily accessible in the ORs where this study was conducted, and it is unclear whether or not knowing cost differences could have an effect on clinician action. Most respondents (84.8%) reported that they would consider using or suggesting to another team member a less expensive device if they were aware of the cost difference between a pair of items with similar clinical outcomes (Fig 4). This willingness to use / suggest using the lower expense item was 91.3% for arthroplasty attendings, in comparison to 82.1% for non- attendings.

**Fig 5:**
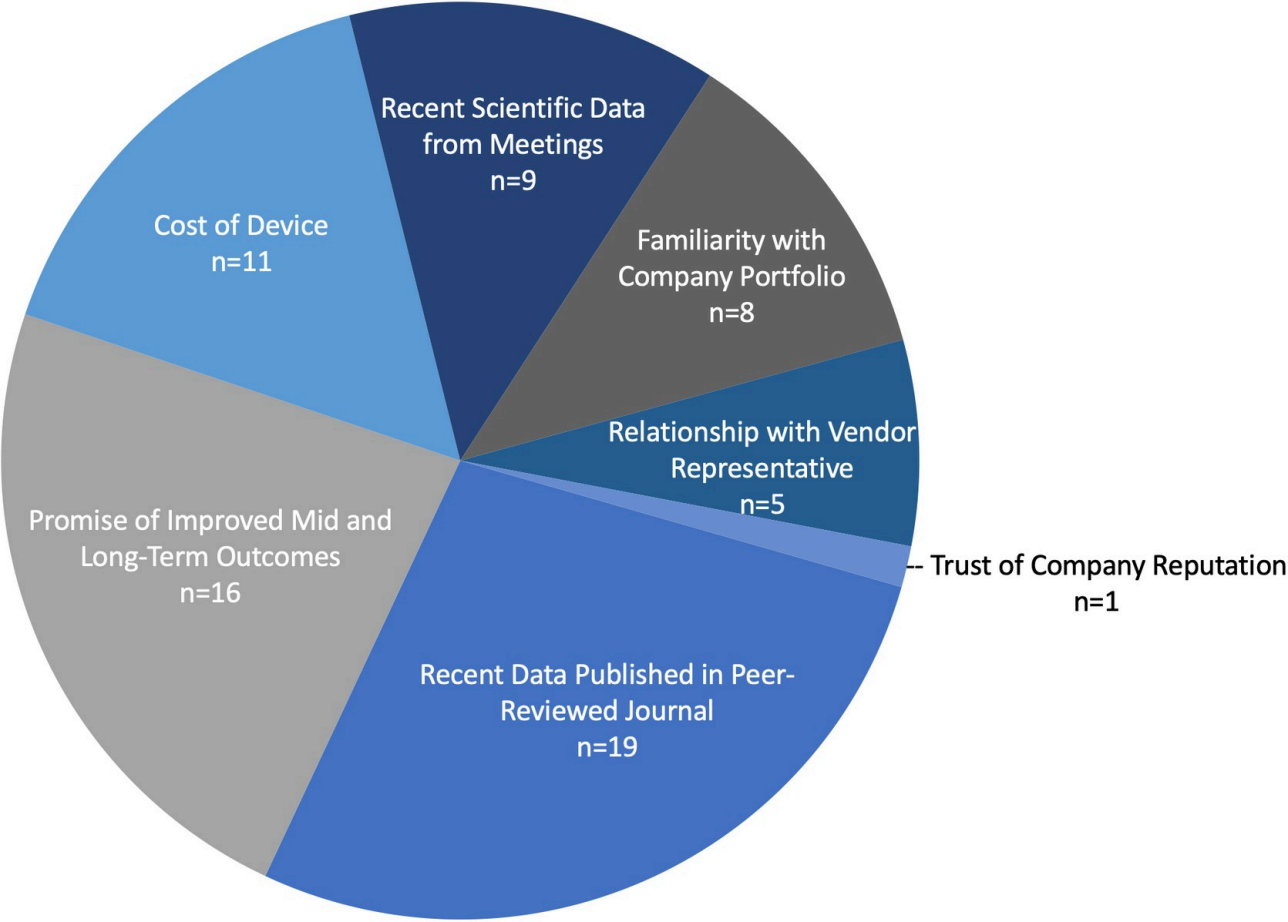
Importance of decision factors in product selection. Importance of decision factors in product selection for arthroplasty attendings.

To that end, arthroplasty attendings asked to select their top three most important decision factors in product selection selected the following: recent data published in peer-reviewed journals (19/23 respondents), promise of improved outcomes (16/23 respondents), and cost of item (11/23 respondents) (Fig 5).

Nonetheless, of the surgical support team, 17.9% of the respondents indicated that they would not feel comfortable suggesting another team member use a less expensive item. Free response queries from these respondents indicated surgeon preference (3/10 respondents), not feeling that it was their place (3/10 responses), not feeling knowledgeable (1/10 respondents) and believing the choice of item is dictated by protocol (1/10 respondents). Half of the respondents (5/10) said they would have a conversation, but not make any recommendation.

## Discussion

The choices of joint arthroplasty materials can have a significant impact on healthcare related costs [14]. Nonetheless, relatively little guidance is provided to surgeons on how to optimize choices and account for costs [14], signaling that value-based healthcare decisions are a flawed process. Further, all on the surgical team have the potential to affect related choices. The current study thus explored the perceptions of cost and clinical value of arthroplasty related products in an academic group using an electronic survey tool.

Overall, the current study found a relatively high percent of respondents to be able to correctly identify the more expensive of paired items- items had average correct of over 90%. Most respondents also perceived the costlier item to be more clinically-valuable. Respondents were less likely to accurately estimate the cost differences between the paired items, with most respondents overestimating the cost differences between items.

Many factors may contribute to physicians’ lack of awareness of costs associated with delivering healthcare. Changes in physician employment arrangements and their relationships with large hospital(s) (systems) may play a role. In private practice or in a physician owned surgical center, physician stakeholders must wear the hat of a business owner, ensuring financial sustainability of their practice in an ever-changing environment [15]. As such, physician stakeholders must be aware of costs associated with their practice to stay afloat [16]. A growing number of newly-minted attending physicians are entering academia or a salaried position in a hospital (system), rather than private practice, often citing the importance of free time [16]. While the burden of administrative duties and paperwork exists for the majority of practicing physicians, this time-intensive burden is often smaller in an employed position. Private practice physicians are often required to be intimately familiar with their billing and insurance claims, while hospital(s) (systems) typically boast entire auxiliary departments dedicated to medical billing and claims [17–20].

Another factor that may contribute to physicians’ lack of awareness of costs associated with delivering healthcare may be the complicated nature of medical device costs for a given hospital or hospital system. Much like creating a drug formulary, hospital administrators often query their clinicians for insight as to which medical devices to procure and stock [9]. While this means that clinicians have significant influence on which devices a hospital (system) purchases, those clinicians are rarely privy to information regarding the per-item costs of those devices. Hospital administrators who negotiate with medical device companies are usually contractually-obligated to keep pricing information confidential [8]. In addition, prices may vary from hospital (system) to hospital (system), since medical device companies typically do not have a single set price for a particular device.

Given the opacity of medical device costs from the perspective of most clinicians, it is understandable that most respondents in our study overestimated the differences in costs between two similar items. Particularly concerning is the implication this may have on clinical practice. Arthroplasty surgeons may avoid or recommend against the use of a certain device to other arthroplasty surgeons, or vice versa, due to a perceived difference in cost. These realities make recommending a particular medical device over another difficult for the price-sensitive clinician.

Although, cost should not be the primary or sole factor in choosing between products, it may be helpful in situations where items are expected to perform similarly. Most respondents (84.8%), especially arthroplasty attendings (91.3%), reported willingness to use a less expensive device (given similar clinical outcomes) if equipped with knowledge of price discrepancies. In fact, arthroplasty attendings cited cost as the third most important factor in deciding which device to select, behind peer-reviewed literature and improved outcomes. Still, of those respondents who were not attendings, 17.9% do not feel comfortable suggesting the less expensive item to arthroplasty attendings, indicating surgeon preference and not feeling that it was their place.

These respondents often cited the hierarchical nature of medicine to explain their hesitance, which speaks to the notorious culture of the OR. Reluctance to challenge authority has resulted in a number of poor outcomes, in medicine and in other professions, such as aviation [21]. Existing literature aimed at identifying barriers to speaking up in the OR have identified a trainees’ lack of communication skills necessary for voicing concerns, a hierarchical climate, the interpersonal communication skills of superiors, and gender [22–26]. In the process of professional identity formation and socialization, trainees, who are lower on the medical totem pole than attending physicians, often must weigh their personal concerns with those of their supervisors [27]. This often results in reluctance to speak up regarding costs of medical devices.

The current study has potential limitations. Survey data may be affected by response bias. Notably, the pairs of items chosen to assess may affect results. Additionally, since the survey was distributed to members of a single academic institution, conclusions from this study may not be generalizable to clinicians at other institutions or in different types of medical practices. Future studies utilizing larger participant pools may be useful to assess differences in cost and value perception based on degree, training level and subspecialty.

## Conclusion

In conclusion, the current study highlights the lack of transparency surrounding costs for medical devices used in TJA. While respondents were generally able to discern which of two similar products is more expensive than the other, respondents often overestimated differences in costs between two similar products, suggesting a dearth of knowledge regarding actual costs of these devices. This may result in a surgeon, consciously or subconsciously, choosing a less expensive product over a more expensive, yet functionally-similar product, or vice versa, or recommending a product to those making purchasing decisions for a hospital (system) that they would not recommend if they had more information on pricing in a standard cost-benefit analysis. The financial impact of this on patients could be substantial, as medical device costs can vary drastically between similar products. Increased transparency and education regarding medical device costs could help to better address and mitigate this knowledge gap.

## Supporting information

S1 Data (XLSX)
